# Supplementary material for: Region-Level Design and Analysis of CRISPR Perturbation Screens with FRACTEL
Source: bioRxiv. 2026 Jul 7:2026.07.06.736794. Preprint. [Version 1] doi: 10.64898/2026.07.06.736794 (PMC13371058; doi:10.64898/2026.07.06.736794)
Supplement: Supplement 17 [file NIHPP2026.07.06.736794v1-supplement-17.pdf]

bioRxiv preprint doi: https://doi.org/10.64898/2026.07.06.736794; this version posted July 7, 2026. The copyright holder for this preprint (which was not certified by peer review) is the author/funder, who has granted bioRxiv a license to display the preprint in perpetuity. It is made available under a CC-BY 4.0 International license.

Supplementary material

List of Supplementary Tables

Table S1	Empirical type I error rates across baseline expression levels

List of Additional Files

File Name	Format	Description
Additional file 1	.tsv	Individual gRNA-gene pair results from SCEPTRE: dCas9-KRAB screen of the MHC locus in induced pluripotent stem cells (iPSCs)
Additional file 2	.tsv	Individual gRNA-gene pair results from SCEPTRE: dCas9-KRAB screen of the MHC locus in neural progenitor cells (NPCs)
Additional file 3	.tsv	Individual gRNA-gene pair results from SCEPTRE: dCas9-KRAB screen of the MHC locus in K562s
Additional file 4	.tsv	Individual gRNA-gene pair results from SCEPTRE: dCas9-p300 screen of the MHC locus in induced pluripotent stem cells (iPSCs)
Additional file 5	.tsv	Individual gRNA-gene pair results from SCEPTRE: dCas9-p300 screen of the MHC locus in neural progenitor cells (NPCs)
Additional file 6	.tsv	Combined intersection set of element-gene pair results from FRACTEL aggregation (k = 0.3n), SCEPTRE's union test, and SCEPTRE's bonferroni correction from the dCas9-KRAB screen of the MHC locus in induced pluripotent stem cells (iPSCs)
Additional file 7	.tsv	Combined intersection set of element-gene pair results from FRACTEL aggregation (k = 0.3n), SCEPTRE's union test, and SCEPTRE's bonferroni correction from the dCas9-KRAB screen of the MHC locus in neural progenitor cells (NPCs)
Additional file 8	.tsv	Combined intersection set of element-gene pair results from FRACTEL aggregation (k = 0.3n), SCEPTRE's union test, and SCEPTRE's bonferroni correction from the dCas9-KRAB screen of the MHC locus in K562s
Additional file 9	.tsv	Combined intersection set of element-gene pair results from FRACTEL aggregation (k = 0.3n), SCEPTRE's union test, and SCEPTRE's bonferroni correction from the dCas9-p300 screen of the MHC locus in induced pluripotent stem cells (iPSCs)
Additional file 10	.tsv	Combined intersection set of element-gene pair results from FRACTEL aggregation (k = 0.3n), SCEPTRE's union test, and SCEPTRE's bonferroni correction from the dCas9-p300 screen of the MHC locus in neural progenitor cells (NPCs)

bioRxiv preprint doi: https://doi.org/10.64898/2026.07.06.736794; this version posted July 7, 2026. The copyright holder for this preprint (which was not certified by peer review) is the author/funder, who has granted bioRxiv a license to display the preprint in perpetuity. It is made available under a CC-BY 4.0 International license.

Additional file 11	.tsv	Combined intersection set of element-gene pair results from FRACTEL aggregation (k = 0.5n), SCEPTRE's union test, and SCEPTRE's bonferroni correction from the dCas9-KRAB screen of the MHC locus in induced pluripotent stem cells (iPSCs)
Additional file 12	.tsv	Combined intersection set of element-gene pair results from FRACTEL aggregation (k = 0.5n), SCEPTRE's union test, and SCEPTRE's bonferroni correction from the dCas9-KRAB screen of the MHC locus in neural progenitor cells (NPCs)
Additional file 13	.tsv	Combined intersection set of element-gene pair results from FRACTEL aggregation (k = 0.5n), SCEPTRE's union test, and SCEPTRE's bonferroni correction from the dCas9-KRAB screen of the MHC locus in K562s
Additional file 14	.tsv	Combined intersection set of element-gene pair results from FRACTEL aggregation (k = 0.5n), SCEPTRE's union test, and SCEPTRE's bonferroni correction from the dCas9-p300 screen of the MHC locus in induced pluripotent stem cells (iPSCs)
Additional file 15	.tsv	Combined intersection set of element-gene pair results from FRACTEL aggregation (k = 0.5n), SCEPTRE's union test, and SCEPTRE's bonferroni correction from the dCas9-p300 screen of the MHC locus in neural progenitor cells (NPCs)
Additional file 16	.txt	List of genes in the MHC locus

bioRxiv preprint doi: https://doi.org/10.64898/2026.07.06.736794; this version posted July 7, 2026. The copyright holder for this preprint (which was not certified by peer review) is the author/funder, who has granted bioRxiv a license to display the preprint in perpetuity. It is made available under a CC-BY 4.0 International license.

Supplementary Tables

n	Baseline expression (UMI)		
	Low	Moderate	High
5	0.0500	0.0496	0.0501
6	0.0496	0.0521	0.0497
7	0.0495	0.0493	0.0499
8	0.0491	0.0498	0.0482
9	0.0486	0.0496	0.0498
10	0.0497	0.0516	0.0513
11	0.0493	0.0502	0.0494
12	0.0502	0.0487	0.0505
13	0.0489	0.0506	0.0498
14	0.0512	0.0505	0.0498
15	0.0514	0.0516	0.0510
16	0.0504	0.0506	0.0490
17	0.0513	0.0499	0.0493
18	0.0489	0.0505	0.0488
19	0.0497	0.0510	0.0495
20	0.0494	0.0496	0.0509

Table S1: Empirical type I error rates across baseline expression levels for varying values of n. Values are estimated from simulations under the null with $\alpha = 0.05$.

bioRxiv preprint doi: https://doi.org/10.64898/2026.07.06.736794; this version posted July 7, 2026. The copyright holder for this preprint (which was not certified by peer review) is the author/funder, who has granted bioRxiv a license to display the preprint in perpetuity. It is made available under a CC-BY 4.0 International license.

Supplementary Figures

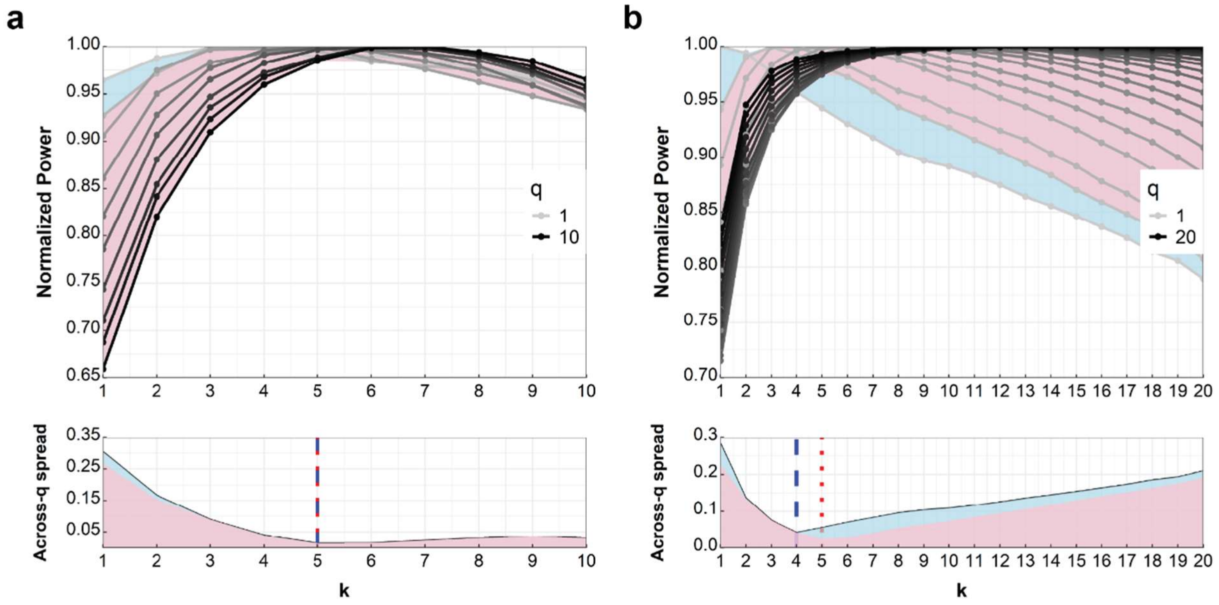

Figure S1: Sensitivity of the optimal bound parameter k. Normalized power curves are shown as in Fig. 2b, with each line scaled by its maximum across k for a fixed number of active gRNAs (q). The shaded region represents the range of normalized power across architectures, and the lower subplot shows the width of this range (max–min) as a function of k. (**a**) Reduced baseline expression. In this setting, the minimum spread occurs near k = 3–4, similar to Fig. 2b. Excluding the single active gRNA case (q = 1) does not materially shift the location of this minimum. However, the spread remains relatively flat for larger k, indicating reduced sensitivity of this criterion for selecting a single preferred value of k under lower baseline expression. **(b)** Increased guide count (n = 20). The minimum spread occurs near k = 4–5, with the precise location depending on whether the q = 1 case is included. In contrast to the n = 10 setting, the spread increases more rapidly for larger k, indicating stronger penalization of higher k values when signal is sparse. These

bioRxiv preprint doi: https://doi.org/10.64898/2026.07.06.736794; this version posted July 7, 2026. The copyright holder for this preprint (which was not certified by peer review) is the author/funder, who has granted bioRxiv a license to display the preprint in perpetuity. It is made available under a CC-BY 4.0 International license.

results suggest that the choice of k becomes more sensitive to both guide count and assumptions about sparsity as n increases.

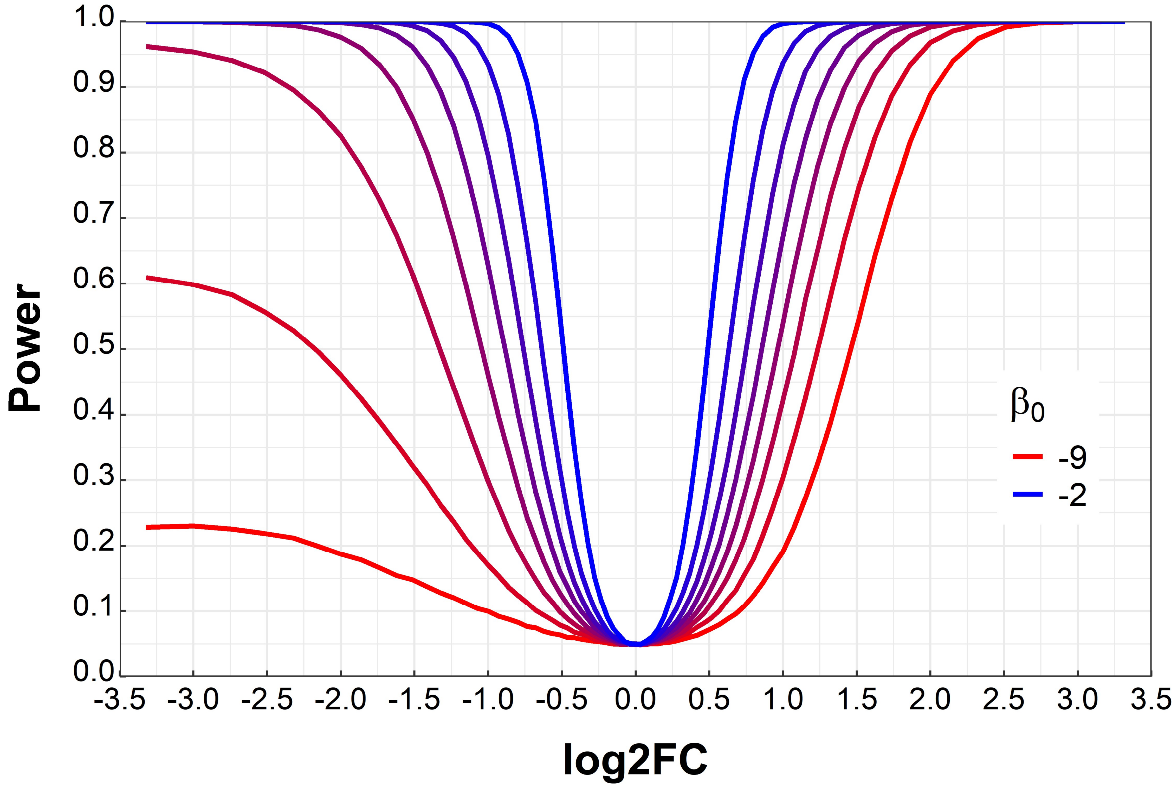

Figure S2: Power curves across different baseline expressions. All curves were simulated with identical parameters except β_0, which ranges from UMI≈0.04 to 40.6. For low values of $\beta_0$repression effects ($log_2\,(FC) < 0$) show clear asymmetry compared to activation effects ($log_2\,(FC) > 0$).This asymmetry disappears as β_0 increases.

bioRxiv preprint doi: https://doi.org/10.64898/2026.07.06.736794; this version posted July 7, 2026. The copyright holder for this preprint (which was not certified by peer review) is the author/funder, who has granted bioRxiv a license to display the preprint in perpetuity. It is made available under a CC-BY 4.0 International license.

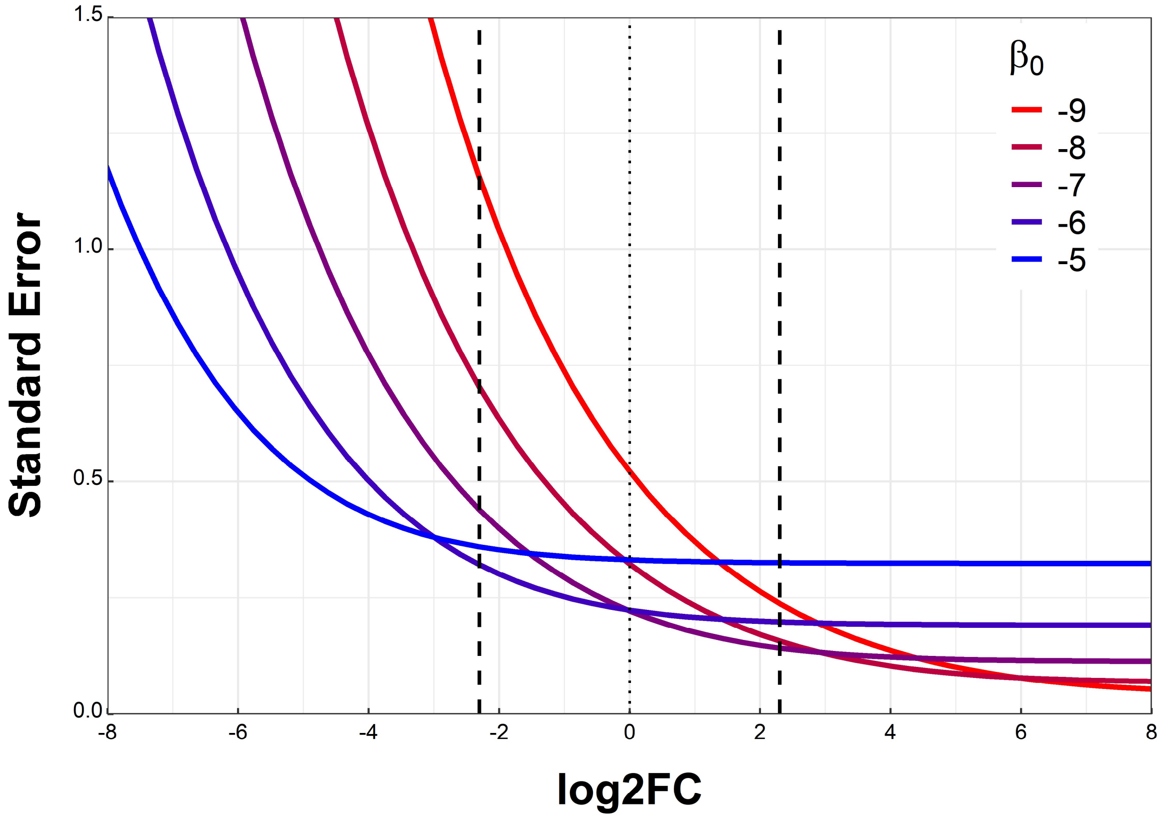

Figure S3: Standard error for varying baseline expressions. While each curve corresponding to a different baseline expression shows that standard error increases as log2FC decreases and plateaus as it increases, the effect becomes more visible in practical effect ranges for lower baseline expression. The vertical black lines correspond to FC=0.1 and FC=10. For all effects on $FC \in (0.1, 10)$, the standard error is effectively constant for UMI >= 2.

bioRxiv preprint doi: https://doi.org/10.64898/2026.07.06.736794; this version posted July 7, 2026. The copyright holder for this preprint (which was not certified by peer review) is the author/funder, who has granted bioRxiv a license to display the preprint in perpetuity. It is made available under a CC-BY 4.0 International license.

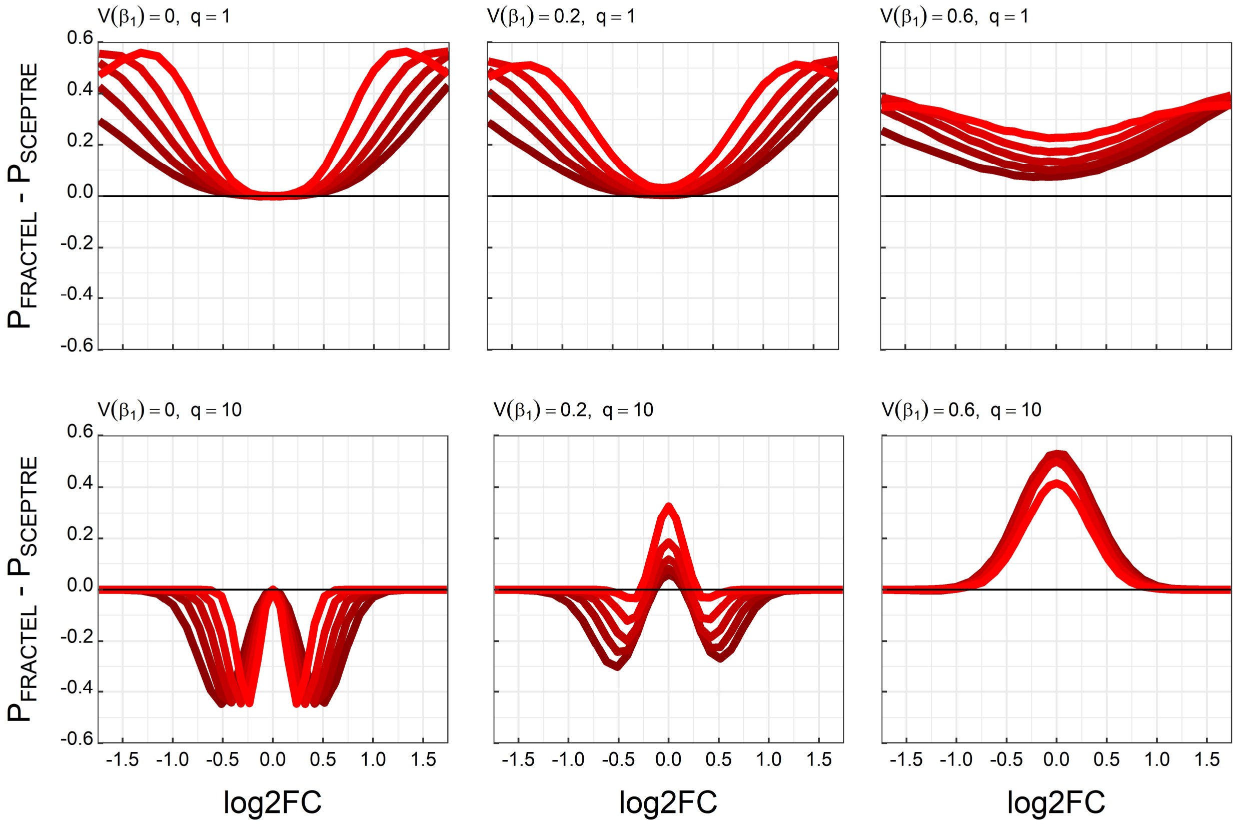

Figure S4: Difference in power between FRACTEL and union-based aggregation for n = 10. Curves show $\Delta Power = P_{FRACTEL} - P_{SCEPTRE}$ across log2 fold-change effect sizes with brighter colors reflecting higher baseline expressions. The left column corresponds to sparse signal (q = 1), and the right column corresponds to dense signal (q = 10). Rows vary the heterogeneity of gRNA effect sizes. The top row shows no variance in effect size (all active guides share the same effect). The middle row introduces moderate heterogeneity, with effect sizes drawn on the log2FC scale from a Normal(β_1, 0.2) distribution. The bottom row shows high heterogeneity, with effect sizes drawn on the log2FC scale from a Normal(β_1, 0.6). When effects are homogeneous and many guides are active (top right), the union method is more powerful across most effect sizes, reflecting efficient aggregation of consistent signal. In contrast, FRACTEL outperforms the union method in sparse settings (q = 1; left column) and as heterogeneity increases

bioRxiv preprint doi: https://doi.org/10.64898/2026.07.06.736794; this version posted July 7, 2026. The copyright holder for this preprint (which was not certified by peer review) is the author/funder, who has granted bioRxiv a license to display the preprint in perpetuity. It is made available under a CC-BY 4.0 International license.

(middle and bottom rows), where variation in effect direction or magnitude reduces the effectiveness of union-based aggregation. Under high variance (bottom row), FRACTEL maintains higher power across nearly all effect sizes, particularly when multiple guides are active.

bioRxiv preprint doi: https://doi.org/10.64898/2026.07.06.736794; this version posted July 7, 2026. The copyright holder for this preprint (which was not certified by peer review) is the author/funder, who has granted bioRxiv a license to display the preprint in perpetuity. It is made available under a CC-BY 4.0 International license.

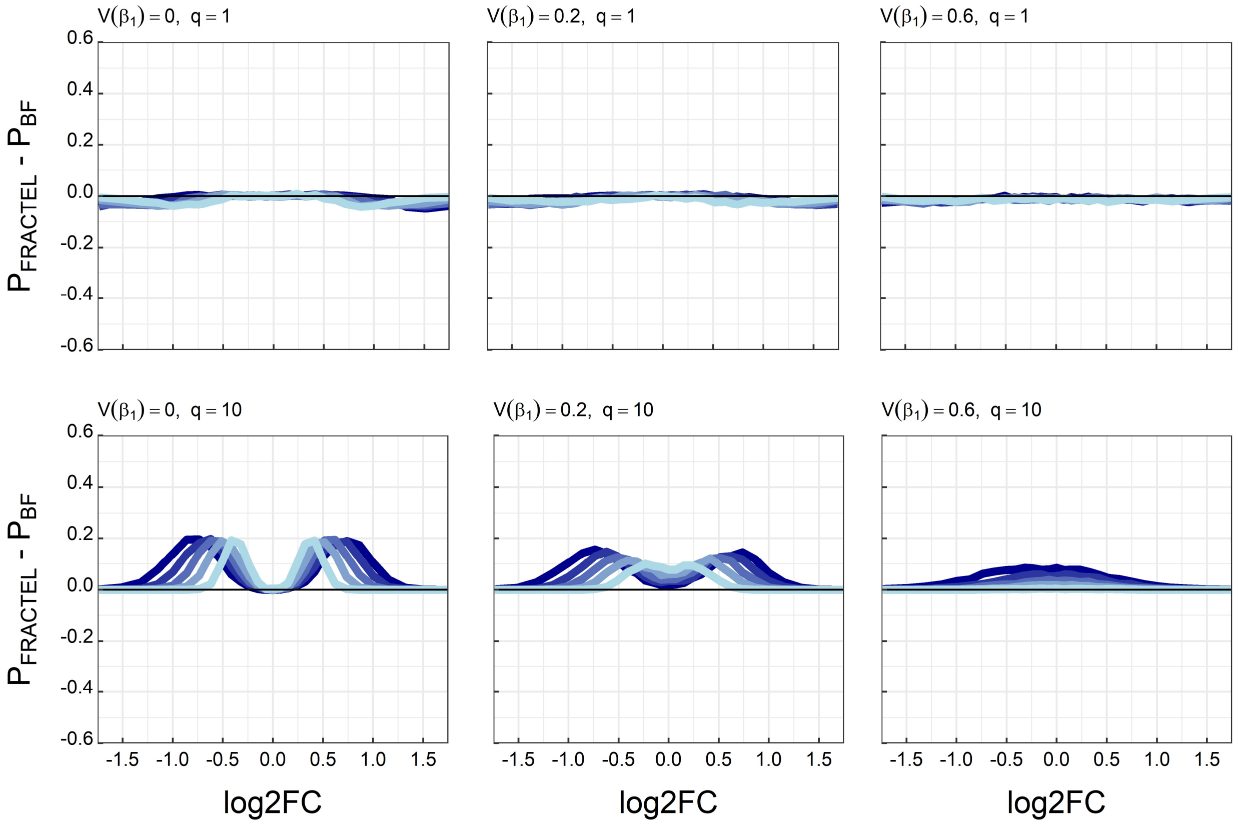

Figure S5: Difference in power between FRACTEL and Bonferroni-corrected minimum p-value aggregation for n = 10. Curves show $\Delta Power = P_{FRACTEL} - P_{BF}$ across log2 fold-change effect sizes with brighter colors reflecting higher baseline expressions. The top row corresponds to sparse signal (q = 1), and the bottom row corresponds to dense signal (q = 10). Columns vary the heterogeneity of gRNA effect sizes. The left column shows no variance in effect size, the middle column introduces moderate heterogeneity, and the right column shows high heterogeneity. For sparse signal (q = 1), FRACTEL and Bonferroni aggregation perform similarly across all levels of effect size variability. For dense signal (q = 10), FRACTEL achieves higher power, reflecting its ability to aggregate signal across multiple active guides. However, this advantage diminishes as effect size variability increases.

bioRxiv preprint doi: https://doi.org/10.64898/2026.07.06.736794; this version posted July 7, 2026. The copyright holder for this preprint (which was not certified by peer review) is the author/funder, who has granted bioRxiv a license to display the preprint in perpetuity. It is made available under a CC-BY 4.0 International license.

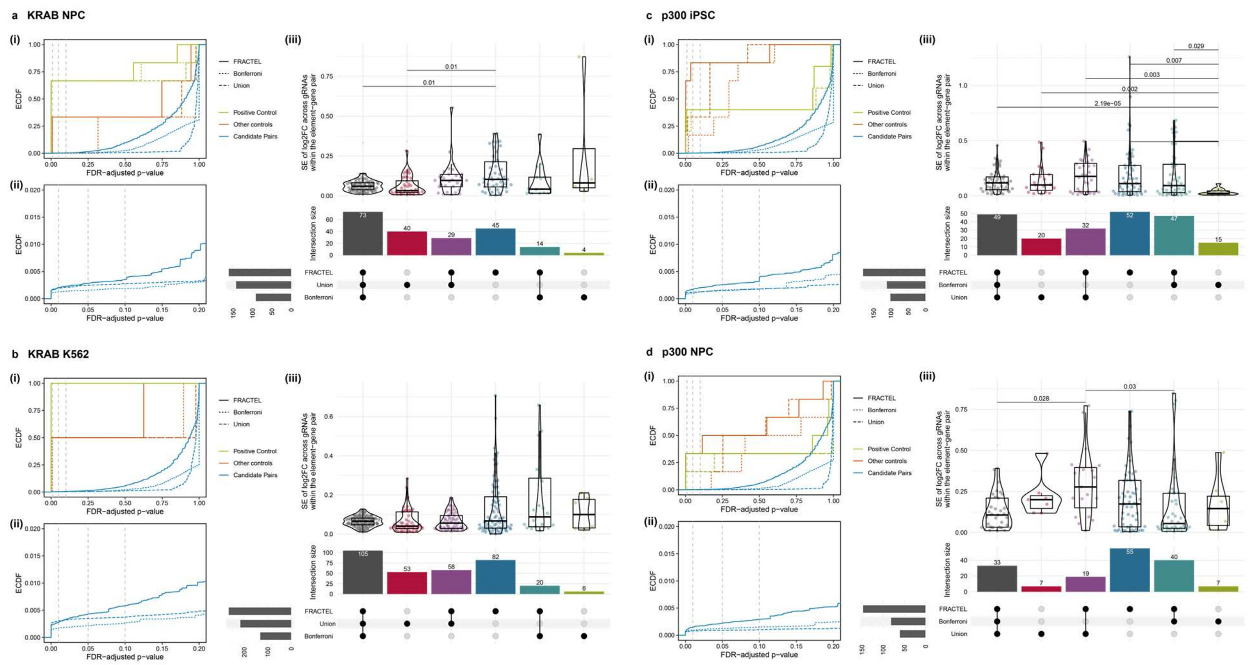

Figure S6. Comparison of aggregation methods across 4 additional screens. Perturb-seq screens of the MHC locus with dCas9-KRAB in **(a)** neural progenitor cells and **(b)** K562s, and with dCas9-p300 in **(c)** induced pluripotent stem cell (iPSCs) and **(d)** neural progenitor cells (NPCs)[1]**. (i)** Empirical cumulative distribution function (ECDF) of the three aggregation methods across FDRs. **(ii)** Plot of ECDF in **(i)** for non-control elements with FDR < 0.2. **(iii)** Comparison of standard error of the effect sizes ($log_2(FC)$) between gRNAs within an element-gene pair, for exclusive intersection sets of significant (FDR < 0.05) elements called by each of the three methods, along with an upset plot to show set sizes. Significant pairwise Mann-Whitney U-tests with a Holm correction are displayed (p_{adj} < 0.05). Not shown is the exclusive intersection of the union and Bonferroni methods (**(a)** n = 0, **(b)** n = 0, **(c)** n = 0, **(d)** n = 1).

bioRxiv preprint doi: https://doi.org/10.64898/2026.07.06.736794; this version posted July 7, 2026. The copyright holder for this preprint (which was not certified by peer review) is the author/funder, who has granted bioRxiv a license to display the preprint in perpetuity. It is made available under a CC-BY 4.0 International license.

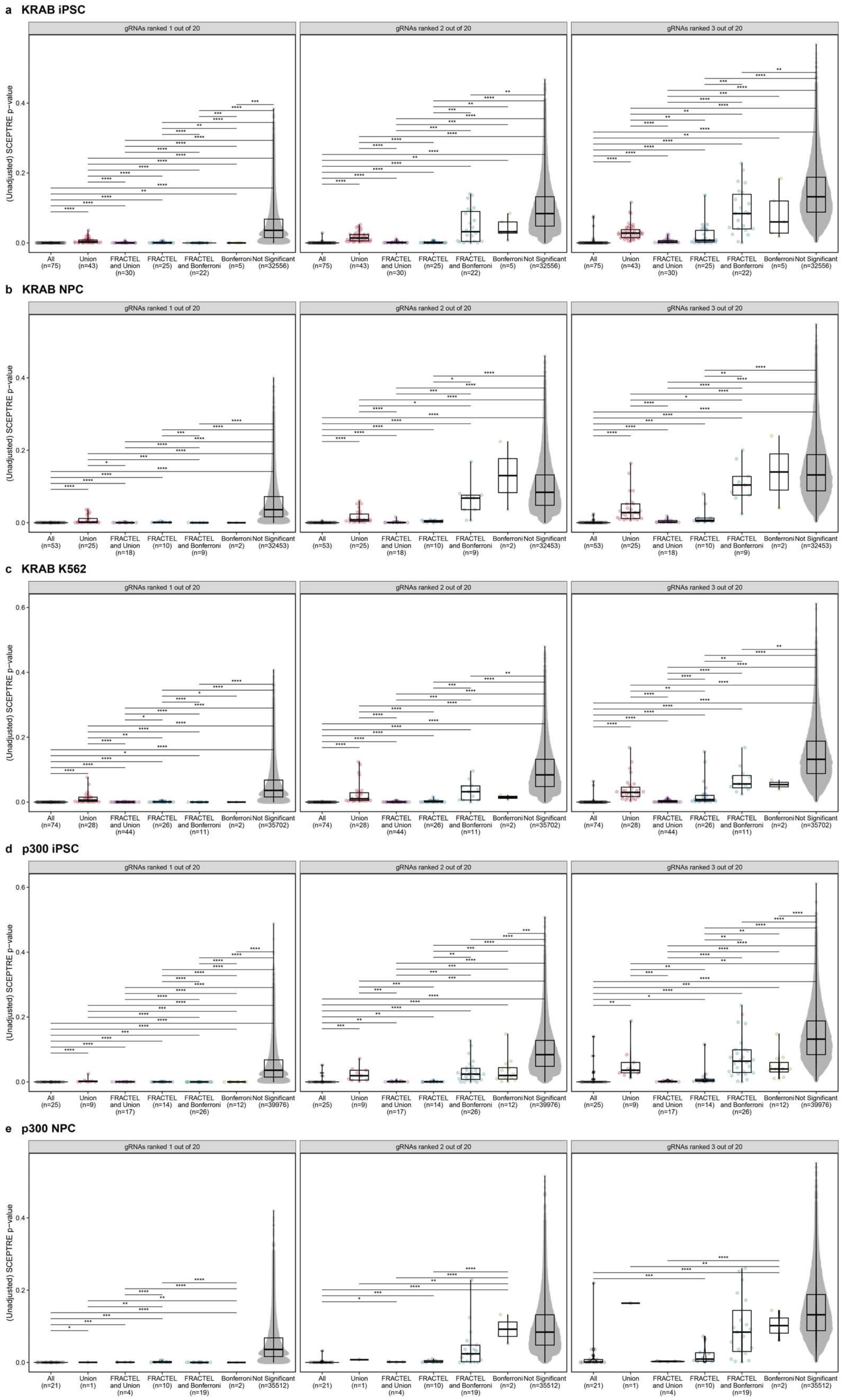

bioRxiv preprint doi: https://doi.org/10.64898/2026.07.06.736794; this version posted July 7, 2026. The copyright holder for this preprint (which was not certified by peer review) is the author/funder, who has granted bioRxiv a license to display the preprint in perpetuity. It is made available under a CC-BY 4.0 International license.

Figure S7. Comparison of aggregation methods' ordered p-values. The first three ordered SCEPTRE gRNA-gene p-values from Perturb-seq screens of the MHC locus with dCas9-KRAB in **(a)** induced pluripotent stem cells (iPSCs) **(b)** neural progenitor cells (NPCs), and **(c)** K562s, and with dCas9-p300 in **(c)** iPSCs and **(d)** NPCs[1]**.** Data were analyzed with SCEPTRE (see Methods) and aggregated to element-gene statistics using either SCEPTRE's Bonferroni method, SCEPTRE's pseudobulked union method, or FRACTEL (k = 0.3n). Only element-gene pairs with 20 complete gRNA-gene pairs are shown. Pairwise Mann-Whitney U-tests were used to compare p-values between exclusive sets called by each of the methods at FDR = 0.05 within each order (significances: **** p< 0.0001, *** p < 0.001, ** p < 0.01, * p < 0.05 after Holm adjustment). Not shown is the exclusive intersection of the union and Bonferroni methods (**(a)** n = 1, **(b)** n = 0, **(c)** n = 0, **(d)** n = 0, **(e)** n = 0).

bioRxiv preprint doi: https://doi.org/10.64898/2026.07.06.736794; this version posted July 7, 2026. The copyright holder for this preprint (which was not certified by peer review) is the author/funder, who has granted bioRxiv a license to display the preprint in perpetuity. It is made available under a CC-BY 4.0 International license.

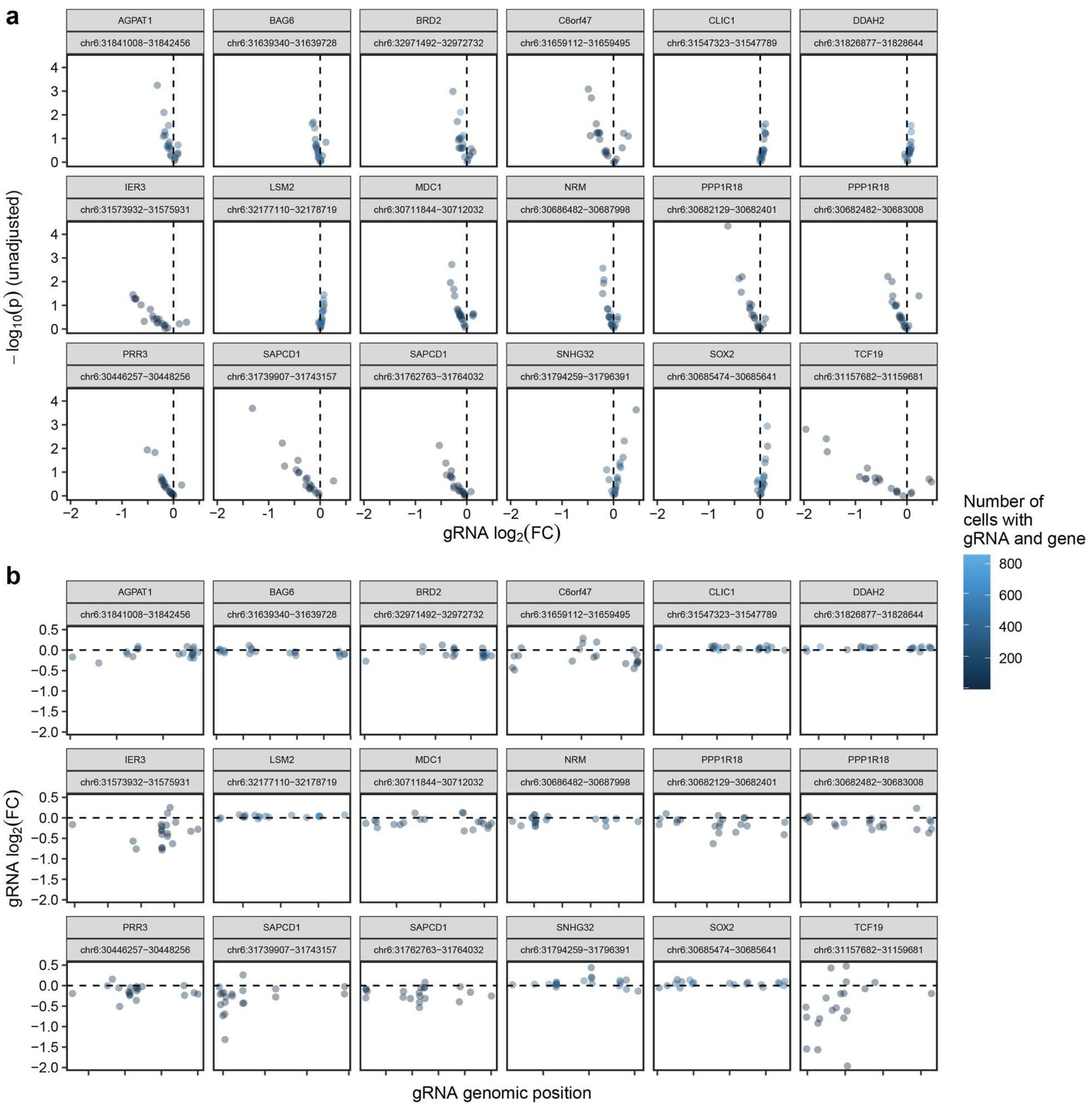

Figure S8: Elements uniquely significant in the union analysis. Examples of some element-gene pairs significant in the union analysis (FDR < 0.05) but not FRACTEL (FDR > 0.2) in a dCas9-KRAB Perturb-seq screen of the MHC locus in neural progenitor cells. Points represent gRNA-gene pairs, colored by the number of cells containing the gRNA and gene, and grouped by element-gene pair. Element-gene pairs are labeled with the affected gene symbol and coordinates of the targeted genomic element. **(a)** Volcano plots

bioRxiv preprint doi: https://doi.org/10.64898/2026.07.06.736794; this version posted July 7, 2026. The copyright holder for this preprint (which was not certified by peer review) is the author/funder, who has granted bioRxiv a license to display the preprint in perpetuity. It is made available under a CC-BY 4.0 International license.

showing the unadjusted significances (-$log_{10}\,p$) and effect sizes ($log_2\,(FC)$) from SCEPTRE for individual gRNA-gene pairs. **(b)** Plots of SCEPTRE gRNA-gene effect sizes ($log_2\,(FC)$), displayed by genomic position across the element within element-gene pairs.

bioRxiv preprint doi: https://doi.org/10.64898/2026.07.06.736794; this version posted July 7, 2026. The copyright holder for this preprint (which was not certified by peer review) is the author/funder, who has granted bioRxiv a license to display the preprint in perpetuity. It is made available under a CC-BY 4.0 International license.

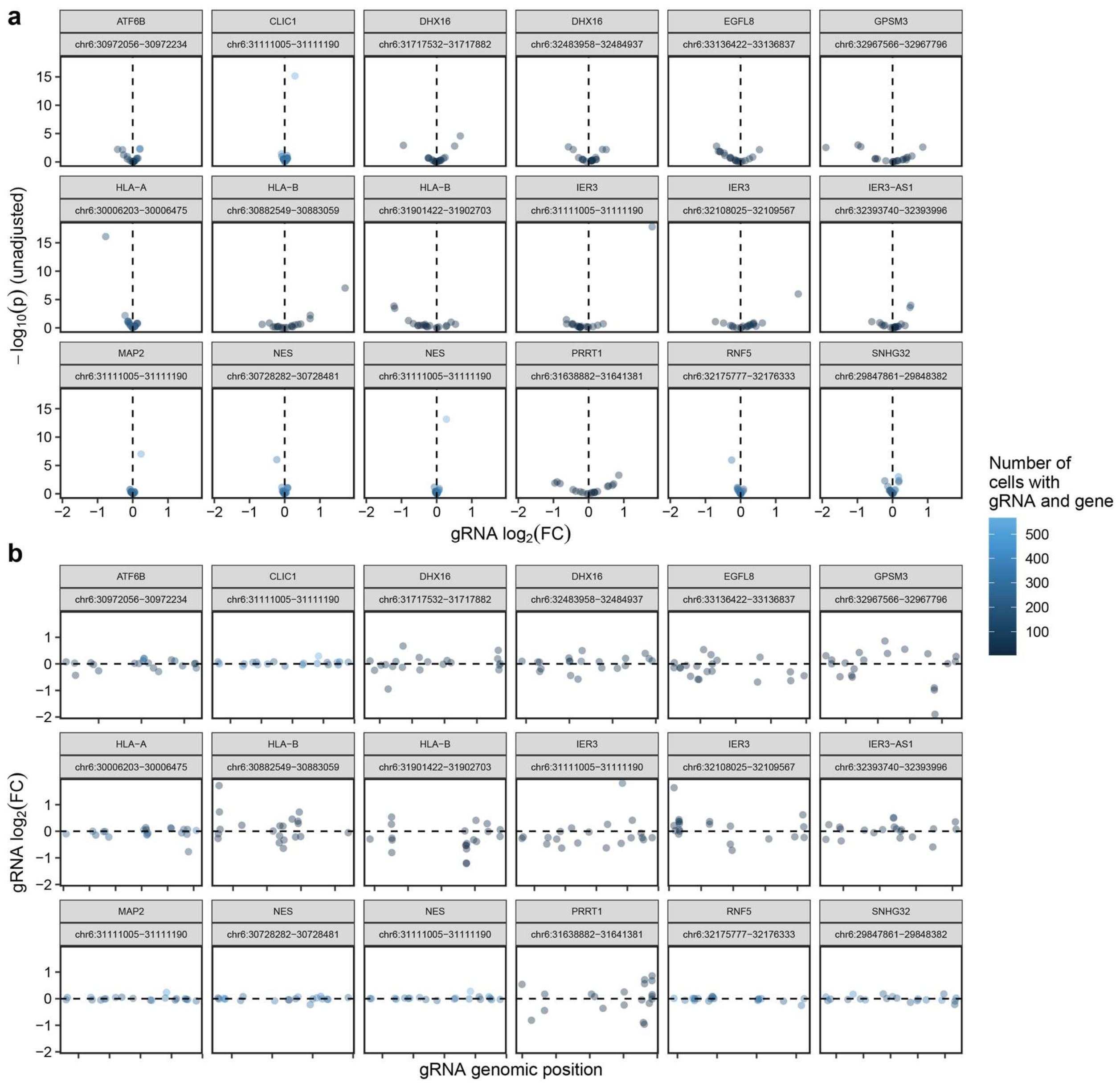

Figure S9: Elements uniquely significant in FRACTEL. Examples of some element-gene pairs significant in FRACTEL aggregation (FDR < 0.05) but not the union analysis (FDR > 0.2) in a dCas9-KRAB Perturb-seq screen of the MHC locus in neural progenitor cells. Points represent gRNA-gene pairs, colored by the number of cells containing the gRNA and gene, and grouped by element-gene pair. Element-gene pairs are labeled with the affected gene symbol and coordinates of the targeted genomic element. **(a)** Volcano

bioRxiv preprint doi: https://doi.org/10.64898/2026.07.06.736794; this version posted July 7, 2026. The copyright holder for this preprint (which was not certified by peer review) is the author/funder, who has granted bioRxiv a license to display the preprint in perpetuity. It is made available under a CC-BY 4.0 International license.

plots showing the unadjusted significances ($-log_{10}\,p$) and effect sizes ($log_2\,(FC)$) from SCEPTRE for individual gRNA-gene pairs. **(b)** Plots of SCEPTRE gRNA-gene effect sizes ($log_2\,(FC)$), displayed by genomic position across the element within element-gene pairs.

bioRxiv preprint doi: https://doi.org/10.64898/2026.07.06.736794; this version posted July 7, 2026. The copyright holder for this preprint (which was not certified by peer review) is the author/funder, who has granted bioRxiv a license to display the preprint in perpetuity. It is made available under a CC-BY 4.0 International license.

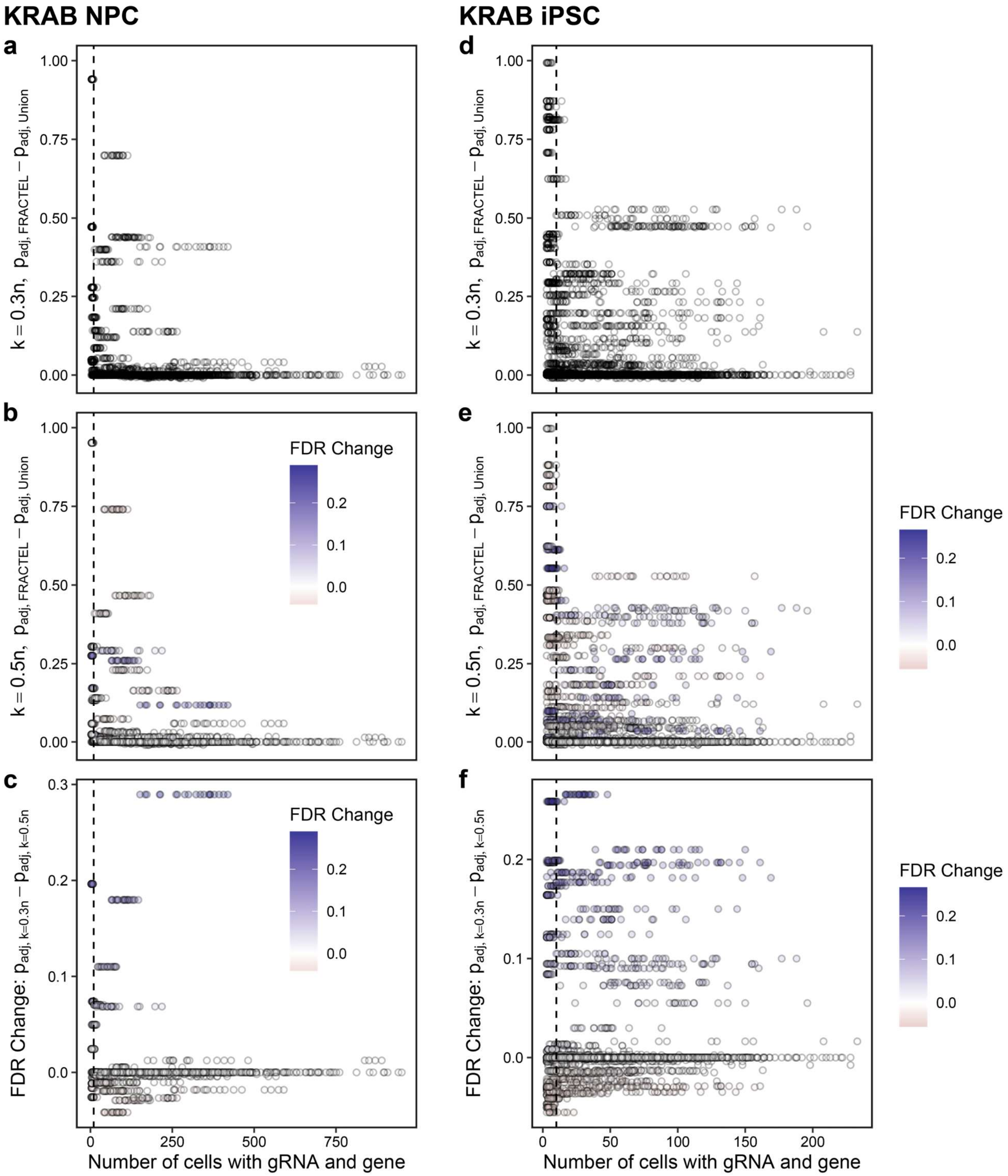

872

Figure S10: Effect of tunable bound on highly significant union elements. Plotted are element-gene pairs with FDR_{Union} < 0.01 from the dCas9-KRAB Perturb-seq screen of the MHC locus in **(a-c)** neural progenitor cells (NPCs) and **(d-f)** induced pluripotent

bioRxiv preprint doi: https://doi.org/10.64898/2026.07.06.736794; this version posted July 7, 2026. The copyright holder for this preprint (which was not certified by peer review) is the author/funder, who has granted bioRxiv a license to display the preprint in perpetuity. It is made available under a CC-BY 4.0 International license.

stem cells (iPSCs). Plotted is the number of cells with the gRNA and gene in the gRNA-level SCEPTRE analysis and **(a,f)** the difference between FRACTEL and union FDRs with $k = 0.3n$, **(b,e)** the difference between FRACTEL and union FDRs with $k = 0.3n$, colored by the FDR change between $k = 0.5n$ and $k = 0.3n$, and **(c,f)** the FDR change between $k = 0.5n$ and $k = 0.3n$, colored identically.

bioRxiv preprint doi: https://doi.org/10.64898/2026.07.06.736794; this version posted July 7, 2026. The copyright holder for this preprint (which was not certified by peer review) is the author/funder, who has granted bioRxiv a license to display the preprint in perpetuity. It is made available under a CC-BY 4.0 International license.

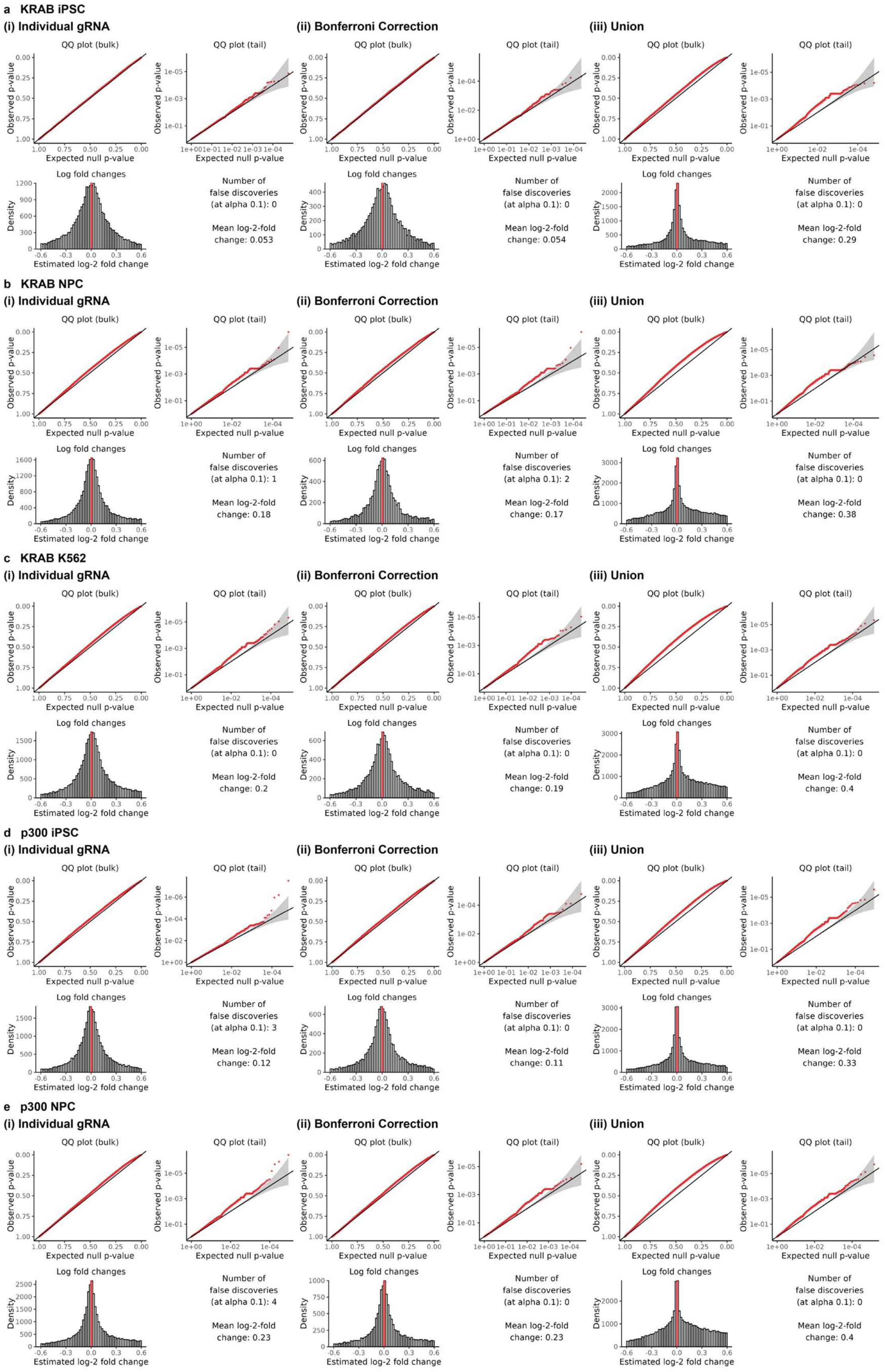

bioRxiv preprint doi: https://doi.org/10.64898/2026.07.06.736794; this version posted July 7, 2026. The copyright holder for this preprint (which was not certified by peer review) is the author/funder, who has granted bioRxiv a license to display the preprint in perpetuity. It is made available under a CC-BY 4.0 International license.

Figure S11: Calibration checks for SCEPTRE analysis. Calibration checks from SCEPTRE for each of the 5 screens Perturb-seq screens of the MHC locus with dCas9-KRAB in **(a)** induced pluripotent stem cells (iPSCs) **(b)** neural progenitor cells (NPCs), and **(c)** K562s, and with dCas9-p300 in **(c)** iPSCs and **(d)** NPCs[48]**.**
